# High-level extracellular production of recombinant nattokinase in *Bacillus subtilis* WB800 by multiple tandem promoters

**DOI:** 10.1186/s12866-019-1461-3

**Published:** 2019-05-07

**Authors:** Zhongmei Liu, Wenhui Zheng, Chunlei Ge, Wenjing Cui, Li Zhou, Zhemin Zhou

**Affiliations:** Key Laboratory of Industrial Biotechnology (Ministry of Education), School of Biotechnology, Jiangnan University, 1800 Lihu Road, Wuxi, 214122 Jiangsu China

**Keywords:** Nattokinase, Tandem promoter, Core promoter region, *Bacillus subtilis*, Recombinant enzyme

## Abstract

**Background:**

Nattokinase (NK), which is a member of the subtilisin family, is a potent fibrinolytic enzyme that might be useful for thrombosis therapy. Extensive work has been done to improve its production for the food industry. The aim of our study was to enhance NK production by tandem promoters in *Bacillus subtilis* WB800.

**Results:**

Six recombinant strains harboring different plasmids with a single promoter (P_*P43*_, P_*HpaII*_, P_*BcaprE*_, P_*gsiB*_, P_*yxiE*_ or P_*luxS*_) were constructed, and the analysis of the fibrinolytic activity showed that P_*P43*_ and P_*HpaII*_ exhibited a higher expression activity than that of the others. The NK yield that was mediated by P_*P43*_ and P_*HpaII*_ reached 140.5 ± 3.9 FU/ml and 110.8 ± 3.6 FU/ml, respectively. These promoters were arranged in tandem to enhance the expression level of NK, and our results indicated that the arrangement of promoters in tandem has intrinsic effects on the NK expression level. As the number of repetitive P_*P43*_ or P_*HpaII*_ increased, the expression level of NK was enhanced up to the triple-promoter, but did not increase unconditionally. In addition, the repetitive core region of P_*P43*_ or P_*HpaII*_ could effectively enhance NK production. Eight triple-promoters with P_*P43*_ and P_*HpaII*_ in different orders were constructed, and the highest yield of NK finally reached 264.2 ± 7.0 FU/ml, which was mediated by the promoter P_*HpaII*_-P_*HpaII*_-P_*P43*_. The scale-up production of NK that was promoted by P_*HpaII*_-P_*HpaII*_-P_*P43*_ was also carried out in a 5-L fermenter, and the NK activity reached 816.7 ± 30.0 FU/mL.

**Conclusions:**

Our studies demonstrated that NK was efficiently overproduced by tandem promoters in *Bacillus subtilis*. The highest fibrinolytic activity was promoted by P_*HpaII*_-P_*HpaII*_-P_*P43*_, which was much higher than that had been reported in previous studies. These multiple tandem promoters were used successfully to control NK expression and might be useful for improving the expression level of the other genes.

**Electronic supplementary material:**

The online version of this article (10.1186/s12866-019-1461-3) contains supplementary material, which is available to authorized users.

## Background

Nattokinase (NK, E.C. 3.4.21.62) was first identified by Sumi et al. from “Natto”, which is a popular traditional Japanese soybean food [1]. NK, as a potent fibrinolytic enzyme, can directly cleave cross-linked fibrin in vitro and inactivate the fibrinolysis inhibitor or catalyze the conversion of plasminogen to plasmin [2, 3]. Studies in rats showed that NK exhibited 5-fold more fibrinolytic activity than that of plasmin [4]. Compared with other thrombolytic reagents, including urokinase, tissue type plasminogen activator (t-PA) and streptokinase, NK has advantages in preventative and prolonged effects, with few side effects and stability in the gastrointestinal tract [5]. The NK gene was cloned and characterized, and protein engineering techniques and site-directed mutagenesis were carried out to improve NK stability [6–10]. The NK enzyme is usually industrially produced by the wild-type *Bacillus subtilis* natto (*B. subtilis* natto) [11].

The species *B. subtilis* is a good host strain for the industrial production of the NK enzyme, as NK was isolated from *B. subtilis* natto. *B. subtilis* is a gram-positive bacterium and is a well-studied host for the expression of heterologous proteins because of its many attractive features [12]. As a model organism, *B. subtilis* is widely used in laboratory studies because it is easy to culture and has a high-level secretory system. In addition, *B. subtilis* is a food-grade safety strain and presents no safety concerns, as reviewed by the U.S. FDA Center. Some efficient expression systems have been constructed to promote the production of homologous and heterologous proteins in *B. subtilis*, because of its well-characterized physiological and biochemical properties and nonpathogenicity [13–15]. *B. subtilis* strains has been engineered as extracellular-protease deficient strains for the overexpression of subtilisin and β-lactamase in *B. subtilis* WB600 [16, 17], the overexpression of staphylokinase and xylanase in *B. subtilis* WB700 [18, 19], and the overexpression of phospholipase C in *B. subtilis* WB800 [20]. In addition, several studies have reported the secretory overexpression of NK in recombinant *B. subtilis* strains [21, 22].

As is well known, the promoter-regulated gene transcription is usually located upstream of the gene. There are two kinds of promoters: the constitutive promoter that is active in all circumstances and the regulated promoter that become active only in response to specific stimulation in the cell. Because the promoter is a crucial aspect of the expression system, many strong promoters have been screened and characterized in *B. subtilis* [23–26]. Recent studies have increasingly focused on the strategy to improve the expression level of recombinant proteins or peptides by the construction of tandem promoters and promoter engineering. Using engineered promoters by altering the − 10 or − 35 region led to a much higher production of recombinant proteins [27, 28]. Widner et al. had studied the gene expression in *B. subtilis* and found that the expression level of the gene could increase by using expression systems that contain two or three tandem promoters in contrast to a single promoter. The study demonstrated that the expression of aprL achieved a high level by combining the mutant *amyQ* promoter with the promoter of the *cry3A* gene [29]. The thermostable 4-α-glucanotransferase from *Thermus scotoductus* was overexpressed in *B. subtilis,* and its productivity was elevated by more than ten-fold when promoted by a dual-promoter system, compared to that of the single HpaII promoter system [30]. Researchers have investigated the strength of single and dual promoters for overexpression of aminopeptidase in *B. subtilis*. In addition, the dual-promoter P_*gsiB*_–P_*HpaII*_ gave the best performance, which was much higher than P_*HpaII*_ and P_*gsiB*_ [31]. The system containing a dual-promoter P_*HpaII*_-P_*amyQ′*_ was found to sustain superior expression of β-cyclodextrin glycosyltransferase in a *B. subtilis* strain (CCTCC M 2016536) [32]. Okegawa and Motohashi successfully expressed the functional ferredoxin-thioredoxin reductase by using a system containing tandem T7 promoters in *Escherichia coli* [33].

In this study, we aimed to increase the secretory expression of NK in *B. subtilis* WB800 by mediating the gene expression promotion by tandem promoters. Six constitutive promoters, P_*HpaII*_, P_*P43*_, P_*BcaprE*_, P_*luxS*_, P_*gsiB*_ and P_*yxiE*_, were selected, and a series of expression cassettes containing single promoters, dual-promoters and triple-promoters was achieved by arranging promoters in different orders. The efficacies of these multiple tandem promoters for controlling the expression of NK are presented.

## Results

### Construction of expression cassettes for overexpression of nattokinase

**Fig. 1:**
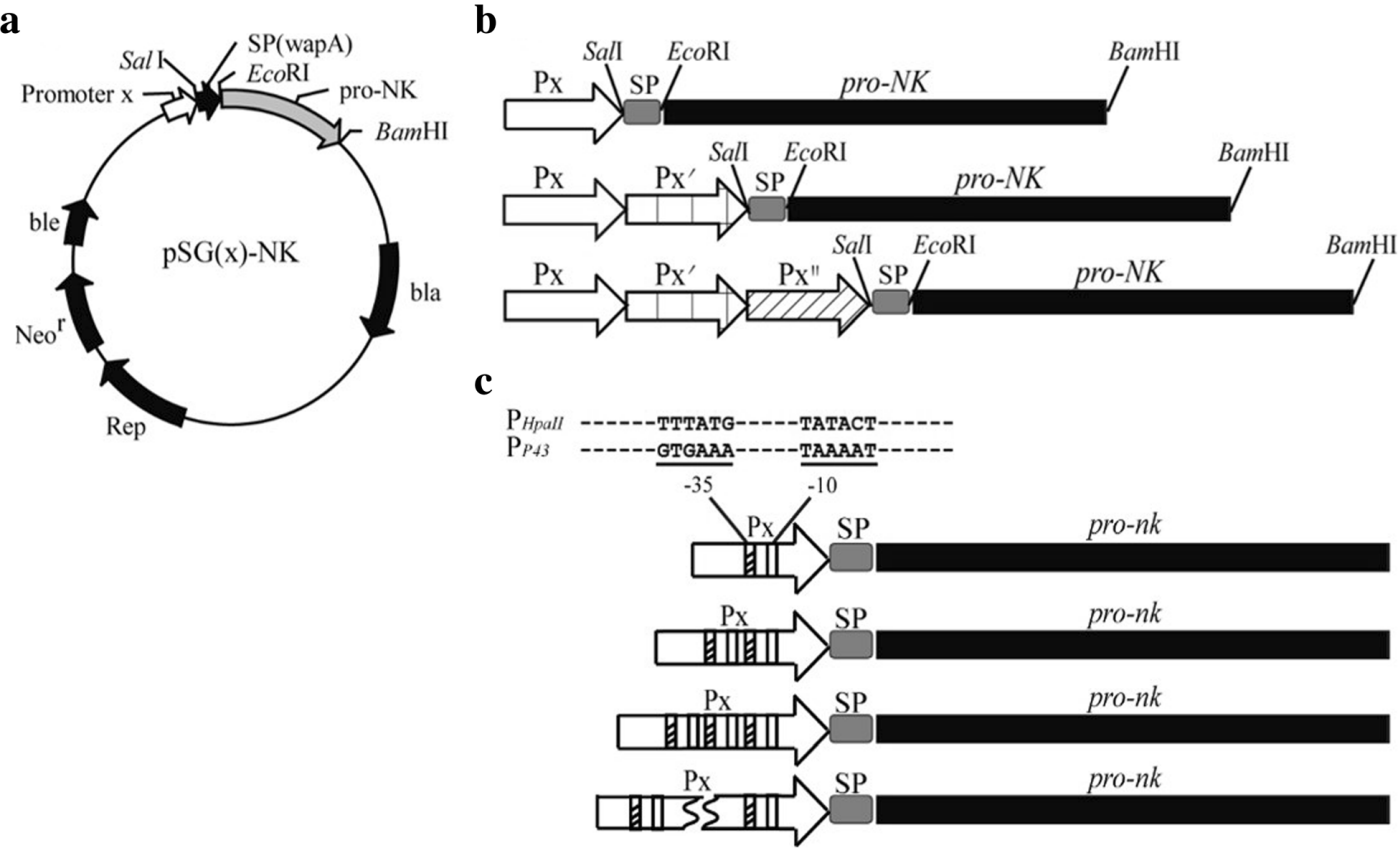
Schematic representation of the expression cassettes. **a** Map of the pSG(x)-NK vectors. All of the expression cassettes were cloned into the pMA0911-*wapA-pro-NK*, and the sites of the relevant restriction enzymes were shown. **b** The schematic diagram of the expression cassettes with tandem promoters. The signal peptide (SP) and the *NK* gene are represented by gray and black, respectively. The promoters, P_X_, are represented by arrows. **c** The expression cassettes with repetitive core regions of promoters. The sequences of core regions (− 35 and − 10) are shown

Six strong and widely used promoters, P_*HpaII*_, P_*P43*_, P_*BcaprE*_, P_*luxS*_, P_*gsiB*_ and P_*yxiE*_*,* were selected as targets for enhancing the production of NK, and their origins and characteristics are listed in Additional file 1: Table S1. The plasmid pSG*-*P_*HpaII*_ was constructed in our previous study [31]. Then, the plasmid pSG*-pro-NK* with no promoter was constructed first, and five promoters were employed to construct the plasmids pSG-P_*P43*_, pSG-P_*BcaprE*_, pSG-P_*luxS*_, pSG-P_*gsiB*_ and pSG-P_*yxiE*_ following the MEGAWHOP method (Fig. 1a).

As shown in Fig. 1b, plasmids harboring multiple promoters in tandem were constructed (pSG-P_X_-P_Y_-P_Z_). These six promoters were further inserted into the downstream region of different promoters to result in fourteen kinds of plasmids in which the NK was controlled by dual-promoters. Based on the NK expression level of recombinant strains under the control of dual-promoters, promoter P43 and HpaII were combined in the pattern of three and four promoters in tandem, and ten different kinds of promoters were successfully obtained.

In addition, another type of tandem promoter (pSG-nCP_X_) was constructed, as shown in Fig. 1c. The core region of the promoter (− 10 and − 35 region) was amplified and linked in tandem repeats. All of the plasmids for the NK expression that was constructed in this study are listed in Table 1.

**Table 1:** Strains and plasmids used in this study

Strains or plasmids	Description	Source	Highest yield of NK (U/mL)
Strains			
*Escherichia coli* JM109	*Rec*A1 *pup*E44 *end*A1 *hsd*R17 *gyr*A96 *rel*A1 *thi*∆(*lac*-*pro*AB) F′[*tra*D36 *pro*AB^+^*lac*I^q^ *lac*Z∆M15]	Lab stock	–
*Bacillus subtilis* WB800	*nprE aprE epr bpr mpr*::*ble nprB*::*bsr* ∆*vpr wprA*::*hyg*	Lab stock	–
Plasmids			
pMA0911-*pro-NK*	shuttle vector for *E. coli*/*B. subtilis*, P_*HpaII*_, SP_*wapA*_, *pro-NK*, Ap^r^, Km^r^,	Lab stock	110.8 ± 5.2
pSG-*pro-NK*	pMA0911-*pro-NK* without promoter P_*HpaII*_	This study	–
pSG-P_*BcaprE*_	pSG-*pro-NK* with promoter P_*BcaprE*_	This study	103.5 ± 4.2
pSG-P_*luxS*_	pSG-*pro-NK* with promoter P_*luxS*_	This study	99.2 ± 3.8
pSG-P_*gsiB*_	pSG-*pro-NK* with promoter P_*gsiB*_	This study	44.6 ± 2.9
pSG-P_*yxiE*_	pSG-*pro-NK* with promoter P_*yxiE*_	This study	20.2 ± 2.0
pSG-P_*P43*_	pSG-*pro-NK* with promoter P_*P43*_	This study	140.5 ± 2.5
pSG-2P_*gsiB*_	pSG-*pro-NK* with promoter P_*gsiB*_-P_*gsiB*_	This study	48.0 ± 2.2
pSG-2P_*BcaprE*_	pSG-*pro-NK* with promoter P_*BcaprE*_-P_*BcaprE*_	This study	120.3 ± 2.4
pSG-2P_*HpaII*_	pSG-*pro-NK* with promoter P_*HpaII*_-P_*HpaII*_	This study	199.4 ± 7.1
pSG-2P_*P43*_	pSG*-pro-NK* with promoter P_*P43*_-P_*P43*_	This study	157.2 ± 4.0
pSG-P_*P43*_*-*P_*HpaII*_	pSG-*pro-NK* with promoter P_*P43*_*-*P_*HpaII*_	This study	231.7 ± 6.0
pSG-P_*HpaII*_-P_*P43*_	pSG-*pro-NK* with promoter P_*HpaII*_-P_*P43*_	This study	210.6 ± 5.2
pSG-P_*BcaprE*_-P_*HpaII*_	pSG-*pro-NK* with promoter P_*BcaprE*_-P_*HpaII*_	This study	175.5 ± 5.0
pSG-P_*HpaII*_-P_*BcaprE*_	pSG*-pro-NK* with promoter P_*HpaII*_-P_*BcaprE*_	This study	0
pSG-P_*yxiE*_-P_*HpaII*_	pSG*-pro-NK* with promoter P_*yxiE*_-P_*HpaII*_	This study	0
pSG-P_*HpaII*_-P_*yxiE*_	pSG*-pro-NK* with promoter P_*HpaII*_-P_*yxiE*_	This study	166.7 ± 2.5
pSG-P_*gsiB*_-P_*HpaII*_	pSG-*pro-NK* with promoter P_*gsiB*_-P_*HpaII*_	This study	164.9 ± 3.0
pSG-P_*HpaII*_-P_*gsiB*_	pSG-*pro-NK* with promoter P_*HpaII*_-P_*gsiB*_	This study	0
pSG-P_*luxS*_-P_*HpaII*_	pSG-*pro-NK* with promoter P_*luxS*_-P_*HpaII*_	This study	77.5 ± 4.0
pSG-P_*HpaII*_-P_*luxS*_	pSG-*pro-NK* with promoter P_*HpaII*_-P_*luxS*_	This study	0
pSG-3P_*HpaII*_	pSG-*pro-NK* with promoter P_*HpaII*_-P_*HpaII*_-P_*HpaII*_	This study	213.3 ± 4.1
pSG-3P_*P43*_	pSG-*pro-NK* with promoter P_*P43*_-P_*P43*_-P_*P43*_	This study	219.2 ± 7.7
pSG-2P_*HpaII*_-P_*P43*_	pSG-*pro-NK* with promoter P_*HpaII*_-P_*HpaII*_-P_*P43*_	This study	264.2 ± 7.0
pSG-P_*P43*_-2P_*HpaIII*_	pSG-*pro-NK* with promoter P_*P43*_-P_*HpaII*_-P_*HpaII*_	This study	47.5 ± 3.1
pSG-P_*HpaII*_-2P_*P43*_	pSG-*pro-NK* with promoter P_*HpaII*_-P_*P43*_-P_*P43*_	This study	199.4 ± 7.1
pSG-2P_*P43*_-P_*HpaII*_	pSG*-pro-NK* with promoter P_*P43*_-P_*P43*_-P_*HpaII*_	This study	149.4 ± 5.0
pSG-P_*HpaII*_-P_*P43*_-P_*HpaII*_	pSG-*pro-NK* with promoter P_*HpaII*_-P_*P43*_-P_*HpaII*_	This study	206.3 ± 7.0
pSG-P_*P43*_-P_*HpaII*_-P_*P43*_	pSG-*pro-NK* with promoter P_*P43*_-P_*HpaII*_-P_*P43*_	This study	182.3 ± 5.6
pSG-4P_*HpaII*_	pSG-*pro-NK* with promoter P_*HpaII*_-P_*HpaII*_-P_*HpaII*_-P_*HpaII*_	This study	200.0 ± 2.6
pSG-4P_*P43*_	pSG-*pro-NK* with promoter P_*P43*_-P_*P43*_-P_*P43*_-P_*P43*_	This study	222.9 ± 4.8
pSG- 2CP_*BcaprE*_	pSG-*pro-NK* with promoter CP_*BcaprE*_-P_*BcaprE*_	This study	120.3 ± 2.4
pSG-2CP_*HpaII*_	pSG-*pro-NK* with promoter CP_*HpaII*_-P_*HpaII*_	This study	200.8 ± 4.6
pSG-3CP_*HpaII*_	pSG-*pro-NK* with promoter CP_*HpaII*_-CP_*HpaII*_-P_*HpaII*_	This study	138.3 ± 3.8
pSG-2CP_*P43*_	pSG-*pro-NK* with promoter CP_*P43*_-P_*P43*_	This study	166.7 ± 5.3
pSG-3CP_*P43*_	pSG-*pro-NK* with promoter CP_*P43*_-CP_*P43*_-P_*P43*_	This study	181.7 ± 6.3
pSG-4CP_*P43*_	pSG-*pro-NK* with promoter CP_*P43*_-CP_*P43*_-CP_*P43*_-P_*P43*_	This study	231.7 ± 8.0
pSG-5CP_*P43*_	pSG-*pro-NK* with promoter CP_*P43*_-CP_*P43*_-CP_*P43*_-CP_*P43*_-P_*P43*_	This study	254.2 ± 5.1

Note: The corresponding highest yield of NK for each construct was detected using the 36-h supernatant

### Expression of nattokinase in *B. subtilis* WB800 with a single promoter

**Fig. 2:**
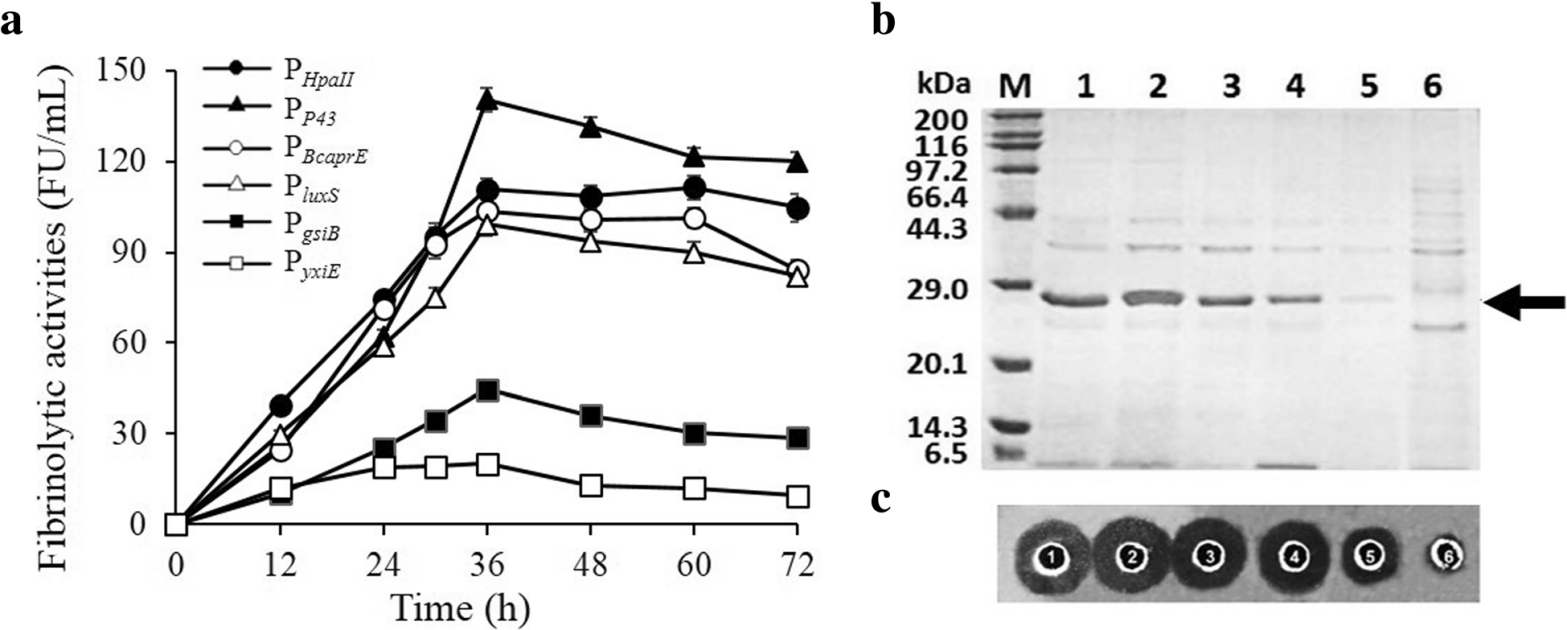
Effects of different single promoters on the overexpression of NK. (**a**) Fibrinolytic activities of NK in the supernatant. The recombinant strains having different single promotes were cultured in TB medium for 72 h with periodical sampling. **b** SDS-PAGE analysis. Recombinant strains having different single promoters were cultured in the TB medium for 36 h, and then the cells and the supernatant culture were separated by centrifugation. Supernatant (15 μL) was loaded into each lane. Lane M: standard marker proteins; Lane 1–6: P_*HpaII*_; P_*P43*_; P_*BcaprE*_; P_*luxS*_; P_*gsiB*_ and P_*yxiE*_. The arrow indicates that the NK bands correspond to 36-h supernatant. **c** Fibrin plate analysis. Transparent zones produced by the enzyme activity of NK and its variants in the supernatant, which was induced for 36 h, were examined by the fibrin plate method, which was conducted at 37 °C for 4 h. 1–6: P_*HpaII*_; P_*P43*_; P_*BcaprE*_; P_*luxS*_; P_*gsiB*_ and P_*yxiE*_

To compare the abilities of those six promoters to promote NK expression, the six strains harboring the different plasmids, pSG-P_*HpaII*_, pSG-P_*P43*_, pSG-P_*BcaprE*_, pSG-P_*luxS*_, pSG-P_*gsiB*_ and pSG-P_*yxiE*_*,* were cultivated in TB medium. The effects of these single promoters on the secretory expression level of recombinant NK were determined by SDS-PAGE and fibrinolytic analysis (Fig. 2). Fibrinolytic activity curves showed that the highest activity was achieved at 36 h (Fig. 2a). The highest yield of NK mediated by P_*HpaII*_ was 110.8 ± 3.6 FU/ml, while the maximum NK activity was 140.5 ± 3.9 FU/ml produced by the strain harboring pSG-P_*P43*_. The expression levels under the control of P_*BcaprE*_ (103.5 ± 4.2 FU/ml) and P_*luxS*_ (99.2 ± 3.8 FU/ml) were similar, second only to the expression under the control of P_*HpaII*_. The promoter P_*yxiE*_ (20.2 ± 2.0 FU/ml) exhibited the lowest expression level of NK among the six promoters, and its promoter strength was only 14% of P_*P43*_. The results of SDS-PAGE and the fibrin plate assay supported the above fibrinolytic analysis results (Fig. 2b and c).

### Effects of different dual-promoter systems on nattokinase expression

**Fig. 3:**
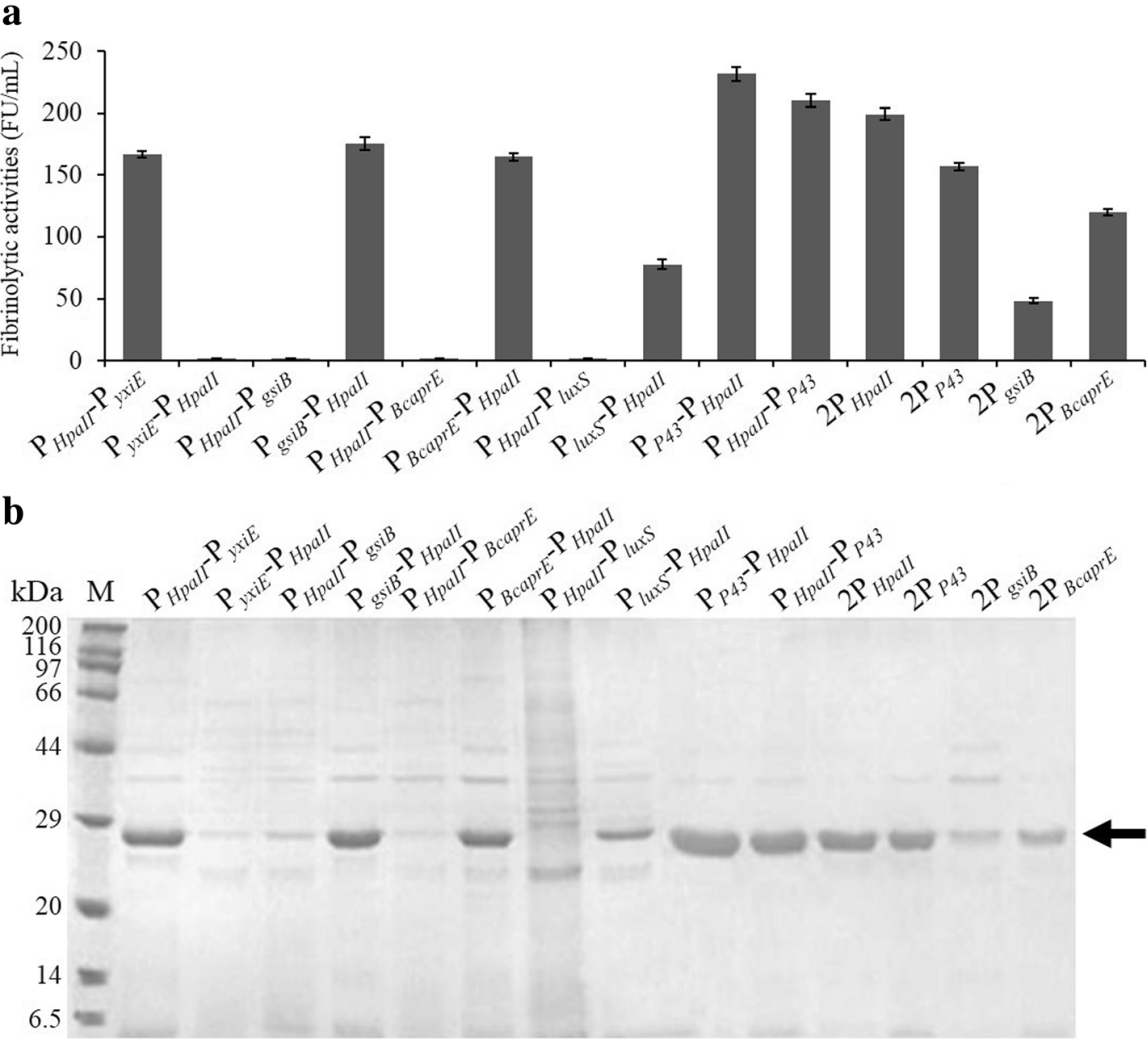
Overproduction of NK under the control of the dual-promoter systems. **a** Fibrinolytic activities of NK in the supernatant. **b** SDS-PAGE analysis. Lane M: standard marker proteins. The position of the NK protein bands is indicated by an arrow. Recombinant strains having different dual-promotes were cultured in the TB medium for 36 h, and then the cells and the supernatant culture were separated by centrifugation

To investigate whether two of these promoters in tandem could enhance NK production, fourteen types of dual-promoters were constructed. The effects of these dual-promoter systems on the expression of recombinant NK were compared by SDS-PAGE and by measuring the fibrinolytic activity (Fig. 3). The NK expression from these dual-promoters containing two of the same promoters was constitutively increased compared with that from a single promoter, such as P_*P43*_-P_*P43*_ (157.2 ± 3.0 FU/ml) compared with P_*P43*_ (140.5 ± 3.9 FU/ml), P_*HpaII*_-P_*HpaII*_ (199.4 ± 4.8 FU/ml) compared with P_*HpaII*_ (110.8 ± 3.6 FU/ml), P_*BcaprE*_-P_*BcaprE*_ (120.3 ± 2.4 FU/ml) compared with P_*BcaprE*_ (103.5 ± 4.2 FU/ml), and P_*gsiB*_-P_*gsiB*_ (48.0 ± 2.2 FU/ml) compared with P_*gsiB*_ (44.6 ± 2.9 FU/ml). These results showed that the experiments involving P_*gsiB*_ in tandem or separately did not exhibit an efficient expression of NK.

Intriguingly, the dual-promoter system containing different promoters showed that the order of two promoters has an important effect on the expression of NK. The NK activity under the control of P_*HpaII*_-P_*yxiE*_ was approximately 166.7 ± 2.5 FU/ml, but the production under the control of P_*yxiE*_-P_*HpaII*_ displayed an obviously opposite effect, in which the expression of NK was undetected (0 FU/ml). Similar results were observed in strains harboring pSG-P_*gsiB*_-P_*HpaII*_ (164.9 ± 3.0 FU/ml) and pSG-P_*HpaII*_-P_*gsiB*_ (0 FU/ml), pSG-P_*BcaprE*_-P_*HpaII*_ (175.5 ± 5.0 FU/ml) and pSG-P_*HpaII*_-P_*BcaprE*_ (0 FU/ml), and pSG-P_*luxS*_-P_*HpaII*_ (77.5 ± 4.0 FU/ml) and pSG-P_*HpaII*_-P_*luxS*_ (0 FU/ml).

However, regardless of how P_*HpaII*_ and P_*P43*_ were arranged in tandem, NK was expressed at a high level in the recombinant strain *B. subtilis* WB800. The NK yield mediated by pSG-P_*P43*_-P_*HpaII*_ reached the highest value (231.7 ± 6.0 FU/ml), which increased by 109% when compared with P_*HpaII*_ and 64.9% when compared with P_*P43*_. The strain harboring pSG-P_*HpaII*_-P_*P43*_ exhibited the second highest expression of 210.6 ± 5.2 FU/ml. The result of the SDS-PAGE analysis (Fig. 3b) was supported by the above results of the fibrinolytic activity. These results showed that NK expression levels under the control of these double promoters were clearly different from each other.

### Effects of different triple-promoters on the nattokinase expression

**Fig. 4:**
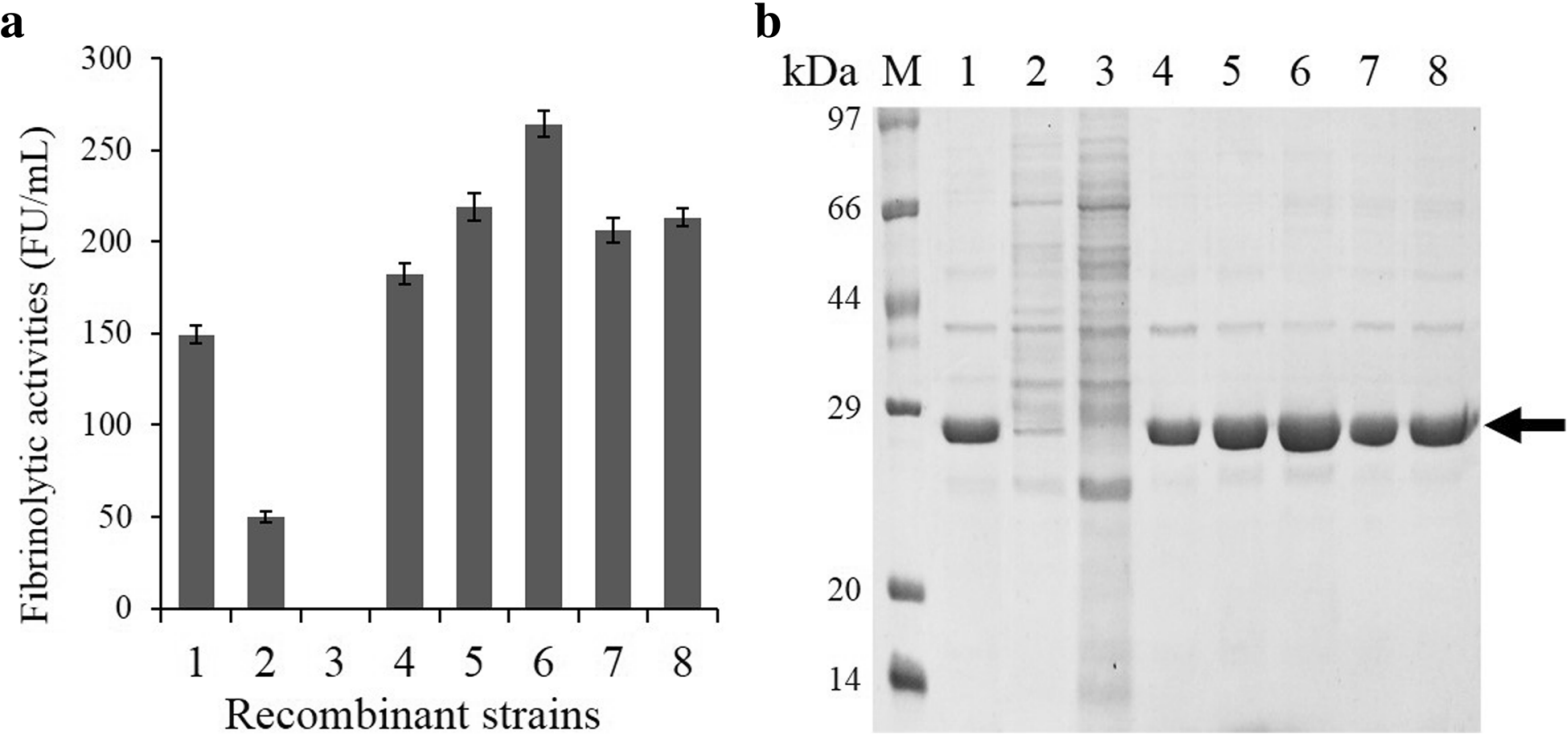
Analysis of the NK production mediated by different triple-promoter systems. **a** Fibrinolytic activities of NK in the supernatant. **b** SDS-PAGE analysis of the culture supernatant. Recombinant strains promoted by different triple-promoters were cultured in the TB medium for 36 h, and then cells and the supernatant culture were separated by centrifugation. Lane 1–8: P_*P43*_-P_*P43*_-P_*HpaII*_, P_*P43*_-P_*HpaII*_-P_*HpaII*_, P_*HpaII*_-P_*P43*_-P_*P43*_, P_*P43*_-P_*HpaII*_-P_*P43*_, P_*P43*_-P_*P43*_-P_*P43*_, P_*HpaII*_-P_*HpaII*_-P_*P43*_, P_*HpaII*_-P_*P43*_-P_*HpaII*_, and P_*HpaII*_-P_*HpaII*_-P_*HpaII*_; Lane M: standard marker proteins. The arrow indicates NK bands

Analysis of NK production showed that promoters P_*HpaII*_ and P_*P43*_ could efficiently promote the expression of NK. To further improve NK production, the expression profiles of eight recombinant strains with three tandem promoters were determined by enzymatic activities and SDS-PAGE (Fig. 4). As shown in Fig. 4a, the NK expression mediated by pSG-P_*HpaII*_-P_*HpaII*_-P_*P43*_ reached the highest activity, 264.2 ± 7.0 FU/ml, which was 14% higher than that under the control of the dual-promoter P_*P43*_-P_*HpaII*_. The triple-promoters P_*HpaII*_-P_*P43*_-P_*HpaII*_ (206.3 ± 7.0 FU/ml) and P_*P43*_-P_*HpaII*_-P_*P43*_ (182.3 ± 5.6 FU/ml) showed similar promoter strengths, and the production that was promoted by both improved considerably compared with the production of P_*HpaII*_ and P_*P43*_. In contrast, P_*P43*_-P_*HpaII*_-P_*HpaII*_ (47.5 ± 3.1 FU/ml) did not exhibit an efficient expression of NK, and P_*HpaII*_-P_*P43*_-P_*P43*_ (0 FU/ml) exhibited no expression of NK. These results indicated that the arrangement of the promoters in tandem has intrinsic effects on the expression level of the target protein.

The NK production of the strain harboring pSG-P_*HpaII*_-P_*HpaII*_-P_*HpaII*_ (213.3 ± 5.1 FU/ml) was increased by 92.2% compared with that under the control of pSG-P_*HpaII*_, and by 7% compared with that under the control of pSG-P_*HpaII*_-P_*HpaII*_. Furthermore, pSG-P_*P43*_-P_*P43*_-P_*P43*_ (219.2 ± 7.7 FU/ml) enhanced the NK production by 55.9% compared with pSG-P_*P43*_, and 39.4% compared with pSG-P_*P43*_-P_*P43*._ The above results of the fibrinolytic activity assays were consistent with those of SDS-PAGE analysis (Fig. 4b).

As the number of promoters increased, the level of NK expression was enhanced up to the triple-promoter. Therefore, we constructed quad-promoter systems, P_*P43*_-P_*P43*_-P_*P43*_-P_*P43*_ and P_*HpaII*_-P_*HpaII*_-P_*HpaII*_-P_*HpaII*_, to test whether the enhancement of NK expression would continue by increasing repetitive promoters. The results in Table 2 showed that the NK activity in the supernatant induced by P_*HpaII*_-P_*HpaII*_-P_*HpaII*_-P_*HpaII*_ decreased slightly. Moreover, the NK production mediated by P_*P43*_-P_*P43*_-P_*P43*_-P_*P43*_ was almost as same as that mediated by P_*P43*_-P_*P43*_-P_*P43*_. These results documented that the expression level of the target protein will not increase unconditionally with the increase in the number of promoters P_*P43*_ or P_*HpaII*_.

**Table 2:** Nattokinase yield under the control of tandem repeats containing whole sequence or core region of P_*HpaII*_ and P_*P43*_

Single	Whole promoter region in tandem		Core promoter region in tandem		
Promoter	Activity (FU/mL)	Promoter	Activity (FU/mL)	Promoter	Activity (FU/mL)
P_*HpaII*_	110.8 ± 5.2	2P_*HpaII*_	199.4 ± 7.1	2CP_HpaII_	200.8 ± 4.6
		3P_*HpaII*_	213.3 ± 4.1	3CP_HpaII_	138.3 ± 3.8
		4P_*HpaII*_	200.0 ± 2.6		
P_*P43*_	140.5 ± 2.5	2P_*P43*_	157.2 ± 4.0	2CP_*P43*_	166.7 ± 5.3
		3P_*P43*_	219.2 ± 7.7	3CP_*P43*_	181.7 ± 6.3
		4P_*P43*_	222.9 ± 4.8	4CP_*P43*_	231.7 ± 8.0
				5CP_*P43*_	254.2 ± 5.1

### Nattokinase expression mediated by core region of P_HpaII_ and P_P43_ in tandem repeats

**Fig. 5:**
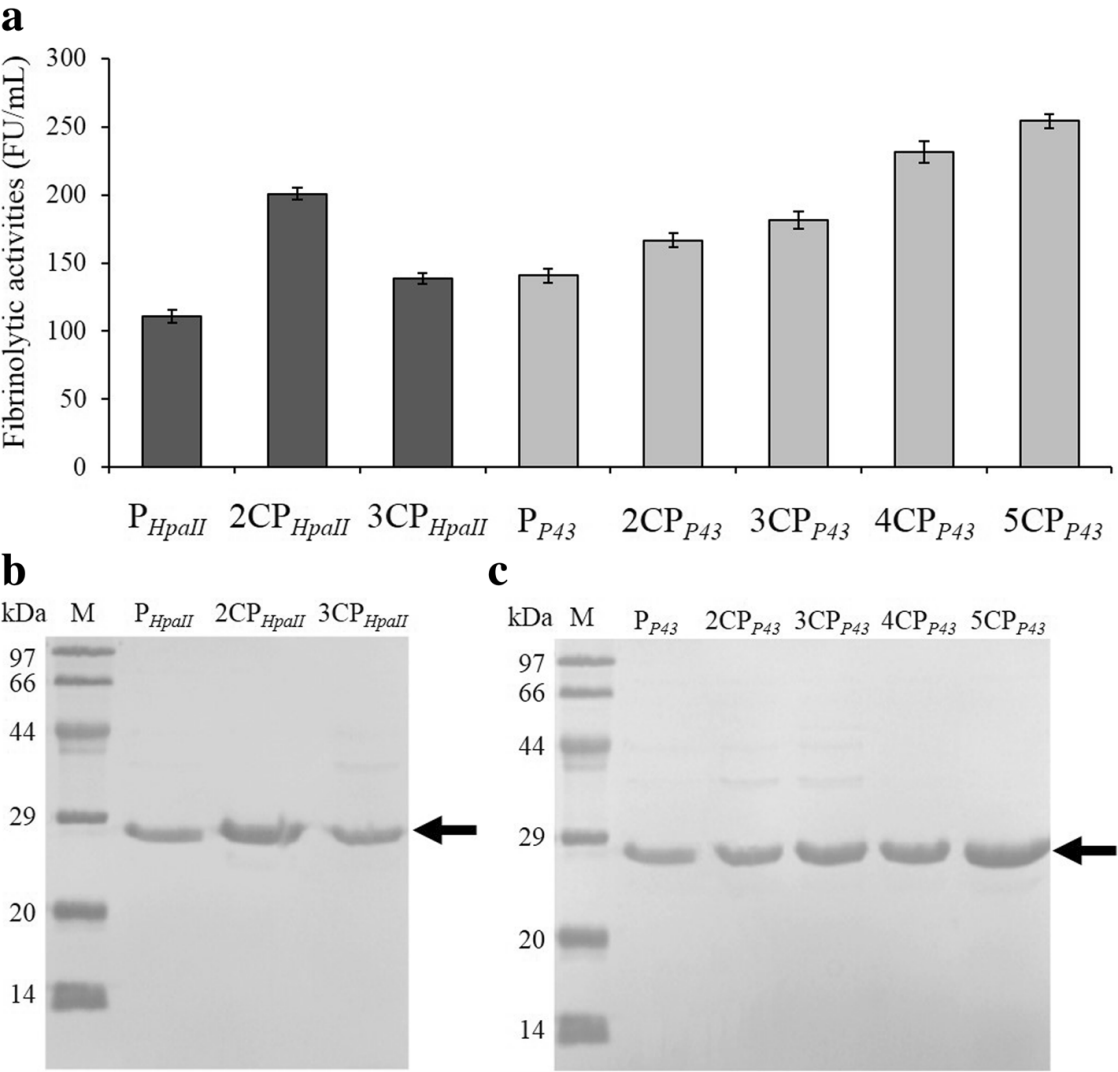
Effects of the multi core regions of P_*HpaII*_ and P_*P43*_ in tandem on NK production. **a** The fibrinolytic activities of NK in the supernatant. Recombinant strains harboring promoters of repetitive core regions were cultured in TB medium for 36 h, and then cells and the supernatant culture were separated by centrifugation. The SDS-PAGE analysis of the NK expression mediated by the repetitive core regions of P_*HpaII*_ (**b**) and P_*P43*_ (**c**). The arrow indicates the NK bands corresponding to the 36-h supernatant, and 15 μL supernatant was loaded into each lane

These two promoters, P_*P43*_ and P_*HpaII*_, had strong abilities to overexpress the recombinant NK in *B. subtilis* WB800. Considering that the length of the promoter affects its expression activity, plasmids harboring the core region of P_*P43*_ or P_*HpaII*_ in tandem repeats (pSG-nCP_X_) were constructed, as shown in Fig. 1c. The NK expression activity of plasmids pSG-nCP_X_ was determined by the fibrinolytic activity and SDS-PAGE analysis (Fig. 5).

As shown in Fig. 5a, the NK production of the strain harboring pSG-2CP_*HpaII*_ (200.8.2 ± 4.6 FU/ml) was increased by 81.2% compared with pSG-P_*HpaII*_. However, the NK production promoted by 3CP_*HpaII*_ (138.3 ± 3.8 FU/ml) decreased by 31.1% compared with that promoted by 2CP_*HpaII*_. It could be seen that the NK expression that was mediated by pSG-5CP_*P43*_ (254.2 ± 5.1 FU/ml) was 80.9% higher than that mediated by pSG-P_*P43*_. The expression level of NK increased with the increase in the number of core regions of P_*P43*_ up to five. The SDS-PAGE analysis showed that the NK expressive quantity in the supernatant produced by pSG-nCP_*HpaII*_ (Fig. 5b) and pSG-nCP_*P43*_ (Fig. 5c) was consistent with the results of the NK activity assay. These results suggested that the core regions of P_*P43*_ and P_*HpaII*_ could produce and enhance the expression level of NK efficiently.

### Scale-up expression of nattokinase in a 5-L fermenter using the strain harboring pSG-P_HpaII_-P_HpaII_-P_P43_

**Fig. 6:**
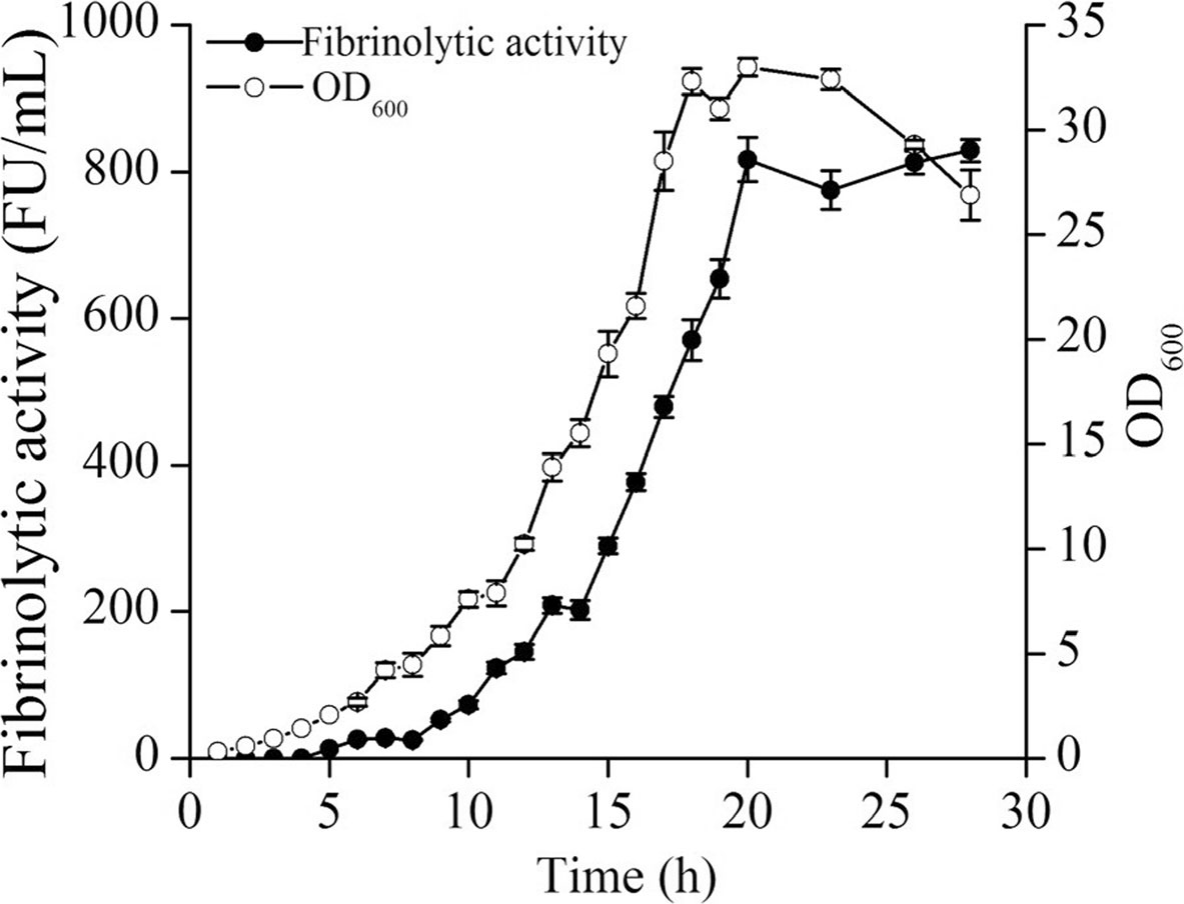
Analysis of fermentation of NK in the recombinant strain harboring pSG-P_*HpaII*_-P_*HpaII*_-P_*P43*_. The fermentation was carried out in a 5-L fermenter, and the cell growth and NK activity were measured by taking a sample every 2 h

Our results indicated that the highest overexpression level of NK was produced by the triple-promoter P_*HpaII*_-P_*HpaII*_-P_*P43*_. Based on the results of the optimization of the cultivation conditions in shaking flask experiments (data not shown), the scale-up of recombinant NK production was completed in a 5-L fermenter using the strain harboring pSG-P_*HpaII*_-P_*HpaII*_-P_*P43*_. The process for the cultivation in the fermenter is shown in Fig. 6. The cell density reached the highest OD_600_ value of 33.0 ± 0.4 at 20 h. Similar to the cell growth, NK production was significantly increased and reached the highest value of 816.7 ± 30.0 FU/ml at 20 h, which was the highest value ever reported. NK production was about two-fold higher in the 5-L fermenter compared to that of the shaking flask experiments. These results indicated that the strain harboring pSG-P_*HpaII*_-P_*HpaII*_-P_*P43*_ had great potential for the industrial production of NK.

## Discussion

Six promoters having high expression strength were selected to overexpress the NK enzyme in *B. subtilis* WB800, and the overexpression of NK mediated by those single promoter systems exhibited significantly different levels. In our study, the highest expression level of NK driven by a single promoter was 140.5 ± 3.9 FU/ml as induced by P_*P43*_. The order of the strength of the six single promoters mediating NK expression in *B. subtilis* was P_*P43*_ > P_*HpaII*_ > P_*BcaprE*_ > P_*luxS*_ > P_*gsiB*_ > P_*yxiE*_. However, Guan et al. reported that the activity of the single promoter P_*P43*_ was lower than that of P_*luxS*_ and P_*yxiE*_ for aminopeptidase expression in *B. subtilis* [31]. In addition, Zhang et al. reported that P_*yxiE*_ exhibited higher expression strengths than P_*P43*,_ both in *B. subtilis* and *E. coli* [23]. The expression level of the target gene is naturally determined by the promoter, signal peptide and host, and many studies have suggested that the effect of the promoter strength on the heterologous expression varies. Our results are consistent with the effect of a promoter varying with the change in the target gene [34]. The growth curves of strains containing a single promoter were approximately same (Additional file 1: Figure S1), and this result confirmed that the expression cassettes with different promoters, not the cell amount, caused the different expression levels of NK.

The NK expression level driven by a dual-promoter P_*P43*_-P_*P43*_ reached 157.2 ± 3.0 FU/ml, which was 10% higher than that induced by the single promoter P_*P43*_. Similar results were observed between the dual-promoter P_*BcaprE*_-P_*BcaprE*_ and the single promoter P_*BcaprE*_ and the dual-promoter P_*gsiB*_-P_*gsiB*_ and the single promoter P_*gsiB*_. However, the strength of the dual-promoter containing two P_*HpaII*_ was 1.8-fold higher than that of the single promoter. The two promoters P_*P43*_ and P_*HpaII*_ exhibited higher promoter activity for NK expression than that of the other promoters. We further carried out the experiments of arranging P_*HpaII*_ and P_*P43*_ by combining three or four promoters in tandem. As shown in Table 2, our results indicated that the NK expression level was not associated with the numbers of tandem repeats of the promoters. The NK production under the control of P_*P43*_-P_*P43*_ was increased by 11.9% compared with that promoted by P_*P43*_, and the NK production mediated by P_*P43*_-P_*P43*_-P_*P43*_ increased by 39.4% compared with that promoted by P_*P43*_-P_*P43*._ However, the NK expression level increased by only 1.7% when promoted by P_*P43*_-P_*P43*_-P_*P43*_-P_*P43*._ Furthermore, NK production decreased under the control of four P_*HpaII*_ in tandem compared with the expression controlled by three tandem promoters. The results were in agreement with studies that suggested that the length of the promoter affects its expression activity [35]. Although the cooperation mechanism of the tandem promoters was not clear, the increased production of NK suggested that this strategy of gene expression based on tandem promoter is an effective way to improve promoter activity.

The core region of a promoter plays an important role in regulating transcription initiation and is the minimal portion of a promoter that is required to properly initiate transcription [36]. To understand the effect of the length of repetitive whole-sequence promoters containing P_*P43*_ or P_*HpaII*_ in tandem on the expression level of NK, a series of promoters with core-region repeats (nCP_*P43*_ and nCP_*HpaII*_) were constructed. The whole sequence of P_*HpaII*_ is 284 bps; however, the core-region sequence of P_*HpaII*_ is only 31 bps. The NK production mediated by 2P_*HpaII*_ and 2CP_*HpaII*_ almost reached the same level, which suggested that the core region of P_*HpaII*_ could efficiently initiate the NK overexpression. However, it was unexpected that the strength of 3CP_*HpaII*_ for NK expression was 35.2% lower than that of 3P_*HpaII*_. Further studies will be needed to explore the difference between the whole sequence and the core regions of P_*HpaII*_ for the level of gene expression. In addition, the whole sequence of P_*P43*_ is 300 bps, and the core region of P_*P43*_ is 29 bps. The NK expression mediated by the whole sequence of P_*P43*_ in tandem increased to that mediated by 4P_*P43*_. The NK production that was initiated by core promoters of P_*P43*_ in tandem gradually increased as the number of core regions increased. It was found that both strong promoters, P_*P43*_ and P_*HpaII*_, have distinct characterization and differential expressions of NK. The analysis of the expression level of NK induced by more core regions of P_*P43*_ in tandem will be carried out.

Obviously different effects on NK production are caused by different arrangements in the dual-promoter system. The promoter is recognized by the σ factor of RNA polymerase to initiate gene transcription. Several σ factors have been defined in *B. subtilis*. It has been reported that σ^A^- and σ^B^-promoters can function cooperatively. The promoter synergism resulting from the double promoters was found only when the σ^B^-promoter was located upstream of the σ^A^-promoter, and the expression level of reporter gene was severely reduced by switching the locations of the σ^A^- and σ^B^-promoters [37]. Since P_*gsiB*_ is σ^B^-dependent (Additional file 1: Table S1), NK production promoted by P_*gsiB*_-P_*HpaII*_ (164.9 ± 3.0 FU/ml) compared with that by P_*HpaII*_ (110.8 ± 3.6 FU/ml) and that by P_*HpaII*_-P_*gsiB*_ (0 FU/ml), suggested that P_*HpaII*_ might be a σ^B^-dependent promoter. Similar phenomena were observed in the results of NK expression mediated by P_*BcaprE*_-P_*HpaII*_ (175.5 ± 5.0 FU/ml) and P_*HpaII*_-P_*BcaprE*_ (0 FU/ml), by P_*luxS*_-P_*HpaII*_ (77.5 ± 4.0 FU/ml) and P_*HpaII*_-P_*luxS*_ (0 FU/ml), predicting that P_*luxS*_ and P_*BcaprE*_ are σ^B^-dependent promoters. Whereas P_*yxiE*_ is σ^A^-dependent (Additional file 1: Table S1), results of NK production promoted by P_*HpaII*_-P_*yxiE*_ (166.7 ± 2.5 FU/ml), compared with that by P_*yxiE*_-P_*HpaII*_ (0 FU/ml) and that by P_*yxiE*_ (20.2 ± 2.0 FU/ml), suggested that P_*HpaII*_ might also be recognized by σ^A^ RNA polymerase. Therefore, promoters P_*HpaII*_ and P_*P43*_ might be recognized by both σ^A^ and σ^B^ RNA polymerases. Our results showed that the NK expression that was promoted by the dual-promoter system makes a large difference, which could be due to the synergistic effect of the double promoters.

Studies have shown that triple-promoters could markedly increase the expression level of heterogeneous genes [29, 38]. We operated by combining both strong promoters in the form of three promoters in tandem. Eight strains harboring a triple-promoter system containing P_*HpaII*_ and P_*P43*_ were generated, from which the NK production showed different levels. Among these 8 strains, one strain harboring the plasmid pSG-P_*HpaII*_-P_*P43*_-P_*P43*_ lost the ability to express NK, and one strain harboring the plasmid pSG-P_*P43*_-P_*HpaII*_-P_*HpaII*_ exhibited low activity of NK expression (47.5 ± 3.1 FU/ml). The other six strains harboring the plasmid containing a triple-promoter exhibited relatively high production of the secreted NK, and the NK expression of four strains were higher than 200 FU/ml. The growth curves of strains containing triple-promoters were approximately the same (Additional file 1: Figure S2), and these results confirmed that the expression cassettes, but not the cell numbers, caused the different levels of NK production with different promoters. On account of the RNA polymerase gene transcription mechanism under the promoter action being very complex, the problem of how to produce this synergy has yet to be further studied. In this study, the highest NK production was mediated by a triple-promoter P_*HpaII*_-P_*HpaII*_-P_*P43*_ and achieved 264.2 ± 7.0 FU/ml, which is much higher than that reported in previous studies [39, 40]. This strain is a potential strain for the industrial production of NK. In addition, the high yield of NK could promote its application in medicine and in supplementary nutrition. A series of plasmids for NK expression in *B. subtilis* were constructed in this study, and they have great potential to be used for NK expression or the expression of other genes in industrial applications. The results of the various initial activities of multiple tandem promoters for NK expression also provide additional information on the synergistic interaction of promoters.

## Conclusions

In this study, we generated and characterized the secretory expression of NK under the control of different promoters, including six single promoters and a series of promoters with the whole sequence or core regions in tandem. The expression level of NK mediated by one of these different promoters led to a remarkable difference in *B. subtilis* WB800. Among the six single promoters, NK production mediated by P_*HpaII*_ and P_*P43*_ exhibited a higher level than the others. The arrangement of these promoters in tandem produced various effects on NK expression. We successively used the triple-promoter P_*HpaII*_-P_*HpaII*_-P_*P43*_ to increase the production of NK to 264.2 ± 7.0 FU/ml in *B. subtilis* WB800, which was the highest expression level ever reported. Our study provided an efficient way to increase NK production in *Bacillus subtilis* based on tandem promoters.

## Materials and methods

### Plasmids, strains and growth conditions

The plasmid pMA0911-*pro-NK*, an *E. coli*/*B. subtilis* shuttle plasmid with the *HpaII* promoter and *wapA* signal peptide, was used to clone and express NK. *E. coli* JM109 served as a host for cloning and plasmid preparation. *B. subtilis* WB800 is deficient in eight extracellular proteases and was used as a host for the NK expression. *Bacillus subtilis* 168 (*B. subtilis* 168) containing the promoter (P_*P43*_) was stored in our laboratory. Transformants were selected on LB agar (0.5% yeast extract, 1% tryptone, 1% NaCl and 2% agar), supplemented with 100 μg/mL ampicillin for *E. coli* JM109 or 50 μg/mL kanamycin for *B. subtilis* WB800. *E. coli* JM109 was cultivated in LB medium supplemented with 100 μg/mL ampicillin. *B. subtilis* WB800 was incubated in TB medium (2.4% yeast extract, 1.2% tryptone, 0.4% glycerol, 17 mM KH_2_PO_4,_ and 72 mM K_2_HPO_4_) additionally containing 0.02% CaCl_2_ and 50 μg/mL kanamycin. All of the strains were cultivated at 37 °C under shaking conditions at 200 rpm. Cell densities were measured using a UV-1800/PC spectrophotometer (MAPADA Instrument Co., Ltd., Shanghai, China). Strains and plasmids used in this study are summarized in Table 1.

### Construction of recombinant plasmids

Primers used in this study were synthesized by Shanghai Sangon Biotech Co., Ltd. and are listed in Table 3. A deficiency of the promoter P_*HpaII*_ from the plasmid pMA0911*-pro-NK* was carried out following megaprimer PCR of the entire plasmid (MEGAWHOP) [41, 42] using primers P0-F and P0-R. The PCR product was digested by *Dpn*I, and the resulting plasmid was transformed into JM109 to yield plasmid pSG*-pro-NK* without a promoter (Table 1).

**Table 3:** Oligodeoxynucleotides used in this study

Primers	Sequence (5′-3′)	Description
P0-F	GGCAAGGGTTTAAAGGTGGAGATTTTTTGAGTGTCGACATGAAAAAAAGAAAGAGGCGAAAC	Upstream for pMA0911-*wapA-pro-NK* construction
P0-R	CCTTTTAAAGTTTCGCCTCTTTCTTTTTTTCATGTCGACACTCAAAAAATCTCCACCTTTAAACC	Downstream for pMA0911-*wapA-pro-NK* construction
P1	GGCAAGGGTTTAAAGGTGGAGATTTTTTGAGTTGATAGGTGGTATGTTTTCGCTTGAAC	Upstream of P_*P43*_
P2	CCTTTTAAAGTTTCGCCTCTTTCTTTTTTTCATGTCGACGTGTACATTCCTCTCTTACCTATAATGG	Downstream of P_*P43*_
P3	GGCAAGGGTTTAAAGGTGGAGATTTTTTGAGTTGCCGAATTCCATGAACGAGACTTAAAACG	Upstream of P_*BcaprE*_
P4	CCTTTTAAAGTTTCGCCTCTTTCTTTTTTTCATGTCGACTCGGTTCCCTCCTCATTTTTATACCAACTTG	Downstream of P_*BcaprE*_
P5	GGCAAGGGTTTAAAGGTGGAGATTTTTTGAGTGATCGTCACAATGCGCCATCAAACCG	Upstream of P_*luxS*_
P6	CCTTTTAAAGTTTCGCCTCTTTCTTTTTTTCATGTCGACGGATCCCACTTTATGGACGCCGCAGTGTCTG	Downstream of P_*luxS*_
P7	GGCAAGGGTTTAAAGGTGGAGATTTTTTGAGTCTATCGAGACACGTTTGGCTGG	Upstream of P_*gsiB*_
P8	CCTTTTAAAGTTTCGCCTCTTTCTTTTTTTCATGTCGACTTCCTCCTTTAATTGGTGTTGGTTGTTGTATTC	Downstream of P_*gsiB*_
P9	GGCAAGGGTTTAAAGGTGGAGATTTTTTGAGTGATCATTTAATTGAAGCGCGCGAAGC	Upstream of P_*yxiE*_
P10	CCTTTTAAAGTTTCGCCTCTTTCTTTTTTTCATGTCGACGCTCTTCCCGCCTTTCGGACTGTGGGTGG	Downstream of P_*yxiE*_
P11	GGGACAGGTAGTATTTTTTGAGAAGATCGTGTACATTCCTCTCTTACCTATAATGG	Downstream for P_*P43*_-P_*HpaII*_
P12	GGCAAGGGTTTAAAGGTGGAGATTTTTTGAGTGATCTTCTCAAAAAATACTACCTGTCCC	Upstream of P_*HpaII*_
P13	GGGACAGGTAGTATTTTTTGAGAAGATCTAAATCGCTCCTTTTTAGGTGGCACAAATGTG	Downstream or P_*HpaII*_-P_*HpaII*_-P_*P43*_

Note: Homology arms of targeting vectors for gene insertions were underlined

The promoter P43 gene was cloned from the genomic DNA of *B. subtilis* 168 with primers P1 and P2. The amplified product was cloned into pSG*-pro-NK* by the MEGAWHOP protocol, yielding plasmid pSG-P_*P43*_. The other single promoters (P_*BcaprE*_, P_*gsiB*_, P_*yxiE*_ and P_*luxS*_) were synthesized by Shanghai Sangon Biotech Co., Ltd. and were employed to construct the plasmids pSG-P_*BcaprE*_, pSG-P_*gsiB*_, pSG-P_*yxiE*_ and pSG-P_*luxS*_, respectively, using the same procedures as for pSG-P_*P43*_.

The six single promoters were further employed to construct 14 kinds of expression cassettes under the control of two promoters in tandem. The plasmid pSG-P_*P43*_-P_*HpaII*_ was constructed by two steps. The fragment of *P43* was amplified from pSG-P_*P43*_ using primers P1 and P11, and then the PCR product was inserted upstream of the promoter *HpaII* in pMA0911-*pro-NK* following the MEGAWHOP protocol, thereby yielding pSG-P_*P43*_-P_*HpaII*_. The same procedures were used to construct the other dual-promoter plasmids.

To construct the triple-promoter plasmid pSG-P_*HpaII*_-P_*HpaII*_-P_*P43*_, the fragment of *HpaII* was amplified from pMA0911-*pro-NK* with primers P12 and P13 and then was inserted into the front of the promoter P_*HpaII*_-P_*P43*_ in pSG-P_*HpaII*_-P_*P43*_ following the MEGAWHOP protocol. The other triple-promoter plasmids and two quad-promoter plasmids (pSG-4P_*HpaII*_ and pSG-4P_*P43*_) were obtained after being treated in the same manner as for pSG-P_*HpaII*_-P_*HpaII*_-P_*P43*_.

The plasmids pSG-nCP_*X*_ harboring the multiple tandem core promoter regions were synthesized by Shanghai RuiDi Biological Technology Co., Ltd. All plasmids were constructed and cloned in *E. coli* JM109 and were sequenced by Shanghai RuiDi Biological Technology Co., Ltd.

### Overexpression of the recombinant nattokinase in *B. subtilis* WB800

Plasmid transformation was carried out according to the method as previously reported [43, 44]. A single colony was inoculated into 10 ml LB medium (including 50 μg/ml kanamycin) and were grown overnight at 37 °C, 200 rpm. The culture was transferred into 100 mL TB medium as a final OD_600_ value of 0.2(v/v), and then was cultivated at 37 °C for 84 h under a shaking condition at 200 rpm for the expression of NK. The supernatant was collected for the following research by centrifugation (10,000 rpm, 5 min) at 4 °C.

### Fed-batch cultivation in 5-L fermenter

Fed-batch cultivations were carried out in a 5-L bioreactor, and the initial medium was 2 L (2% glycerol, 2% soybean peptone, 0.1% NaH_2_PO_4_, 0.2% Na_2_HPO_4_, 0.02% CaCl_2_, and 0.05% MgSO_4_) containing 50 μg/ml kanamycin. The pre-inoculum culture was 50 mL TB medium including 50 μg/mL kanamycin, which was incubated at 37 °C under a shaking condition at 200 rpm. After 12 h, the culture was inoculated into the 5-L fermenter. The inoculation volume was 8%. The cultivated condition was maintained at 37 °C, and the dissolved oxygen (DO) was performed above 30% under the control of the inlet air and the exponential feeding of glycerol and soybean peptone. During the cultivation process, the pH was controlled at 7.0 through the automatic addition of 50% ammonium solution. Samples were taken every 2 h.

### Fibrin plate analysis

A qualitative analysis of the fibrinolytic activity was carried out according to the fibrin plate method [45]. In brief, 10 ml agarose solution (1%) and 10 ml bovine fibrinogen solution (1.8 mg/ml in 50 mM Tris-HCl buffer) were incubated separately at 60 °C, and 10 U thrombin was added into the agarose solution and mixed. The agarose solution and fibrinogen were mixed, and the plate was put at room temperature for 2 h to form fibrin clots. Holes were made in the fibrin plate, and 40 μl enzyme was added in each hole. The fibrin plates were placed at 37 °C for 4 h to detect the fibrinolytic activity.

### Fibrinolytic activity determination

The fibrinolytic activity was determined using the method described by the Japan Nattokinase Association (http://j-nattokinase.org/jnka_nk_english.html). In brief, 1.4 mL Tris-HCl (0.05 M, pH 8.0) and 0.4 mL fibrinogen solution (0.72%) were pre-incubated in a 37 °C water bath for 5 min. Thereafter, 0.1 mL thrombin solution was added, followed by the addition of 0.1 mL diluted sample after 10 min. The mixture was incubated at 37 °C for an hour. Finally, trichloroacetic acid solution (0.2 M) was added and incubated at 37 °C for 20 min to stop the reaction. The supernatant was transferred into a microtest tube after centrifugation (12,000 rpm, 10 min), and the absorbance of the supernatant at 275 nm was read and recorded. One unit (1 FU) was defined as the amount of the enzyme that increased the absorbance of the filtrate at 275 nm by 0.01 per minute. The analysis of fibrinolytic activity was independently carried out in triplicate, and the data are presented as the mean ± s.d.

### SDS-PAGE analysis

Samples were incubated at room temperature for 30 min with 5 × SDS-PAGE loading buffer and protease inhibitor PMSF (phenylmethane sulphonyl fluoride). Then, the samples were heated at 100 °C for 5 min and were applied into 12% SDS-PAGE with 5% stacking gels. Finally, the gels were stained by Coomassie Blue R-250.

## Additional file

Additional file 1: **Table S1** Characterization of single promoters used for the NK production. **Figure S1** The growth curves of recombinant strains harboring different plasmids with a single promoter. **Figure S2** The growth curves of recombinant strains containing a triple-promoter. (DOCX 297 kb)

## Abbreviations

LB: Luria-Bertani medium
NK: Nattokinase
P_*BcaprE*_: *aprE* promoter
P_*gsiB*_: *gsiB* promoter
P_*HpaII*_: *HpaII* promoter
P_*luxS*_: *luxS* promoter
P_*P43*_: *P43* promoter
P_*yxiE*_: *yxiE* promoter
SDS-PAGE: Sodium dodecyl sulfate-polyacrylamide gel electrophoresis
SP: The wapA signal peptide
TB: Terrific Broth medium
