# Supplementary material for: High-level extracellular production of recombinant nattokinase in Bacillus subtilis WB800 by multiple tandem promoters
Source: BMC Microbiol. 2019 May 7;19:89. doi: 10.1186/s12866-019-1461-3 (PMC6505213; doi:10.1186/s12866-019-1461-3)
Supplement: Supplementary file 1 — Table S1 Characterization of single promoters used for the NK production. Figure S1 The growth curves of recombinant strains harboring different plasmids with a single promoter. Figure S2 The growth curves of recombinant strains containing a triple-promoter. (DOCX 297 kb) [file 12866_2019_1461_MOESM1_ESM.docx]

*BMC Microbiology*

**High-level extracellular production of recombinant nattokinase in *Bacillus subtilis* by multiple tandem promoters**

Zhongmei Liu^*^, Wenhui Zheng, Chunlei Ge, Wenjing Cui, Li Zhou, Zhemin Zhou

Key Laboratory of Industrial Biotechnology (Ministry of Education), School of Biotechnology, Jiangnan University, 1800 Lihu Road, Wuxi, Jiangsu 214122, China

* Corresponding author

Zhongmei Liu, Ph. D. (Associate Professor)

School of Biotechnology, Jiangnan University, Wuxi, Jiangsu 214122, China.

Tel: +86-510-85325210, E-mail: [zliu@jiangnan.edu.cn](mailto:zliu@jiangnan.edu.cn)

**
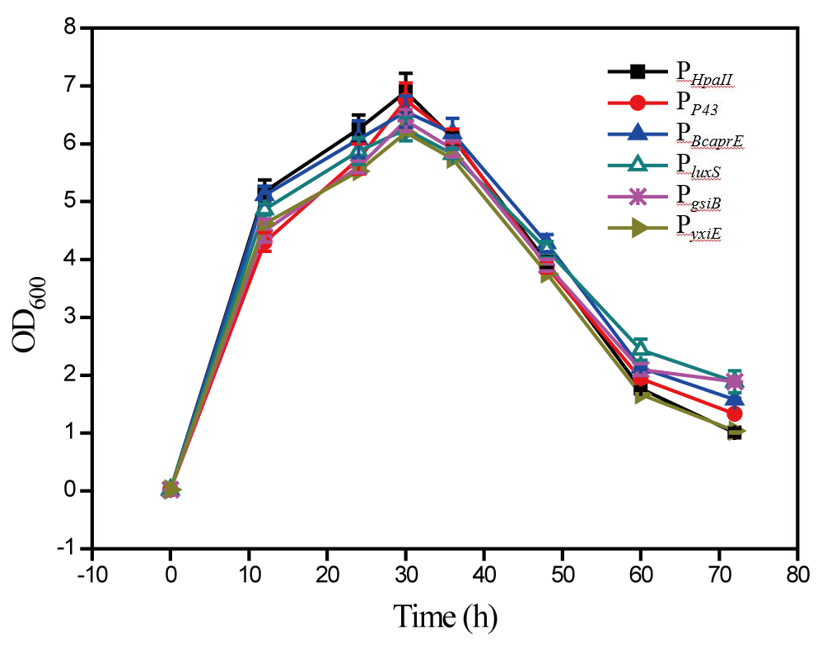
**

**Figure S1. The growth curves of recombinant strains harboring different plasmids with a single promoter.** The recombinant strains were cultured in TB medium for more than 72 h with periodical sampling.

**
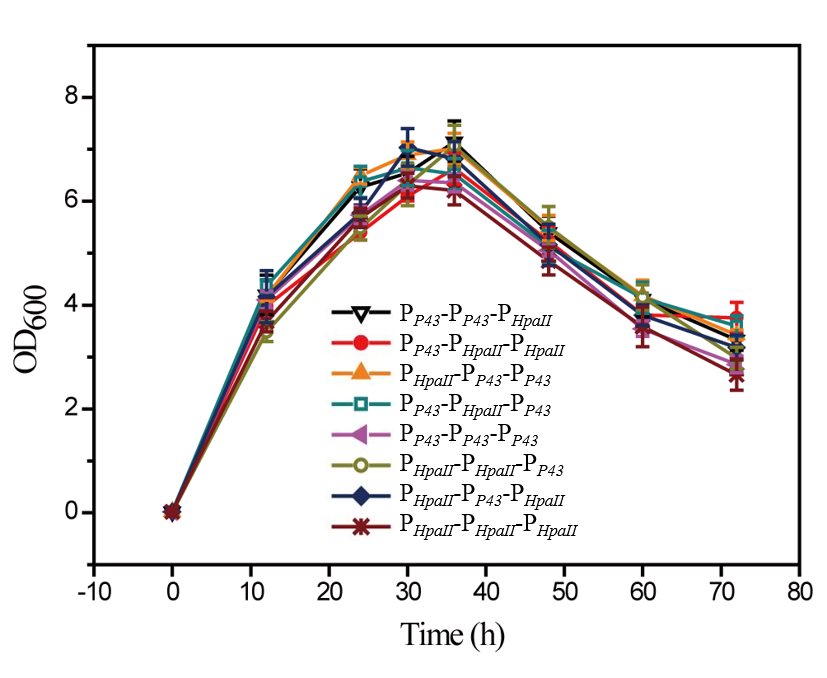
**

**Figure S2. The growth curves of recombinant strains containing a triple-promoter**. The recombinant strains were cultured in TB medium for more than 72 h with periodical sampling.

**Table S1 Characterization of single promoters used for the NK production**

| Promoter | Source | Properties | Reference |
| --- | --- | --- | --- |
| P*_HpaII_* | *Staphylococcus aureus* | Cloned from plasmid pUB110, and stimulate synthesis of the counter-clockwise RNA | DNA. 1986, 5:219-225. |
| P*_P43_* | *Bacillus subtilis* | Recognized by both σ^A^ and σ^B^ RNA polymerase | Appl Environ Microbiol. 2005, 71:4101-4103. |
| P*_BcaprE_* | *Bacillus clausii* | Promoter of *AprE* (*aprE*) gene from *Bacillus clausii* EHY L2 strain; GenBank: HQ849486.1 | J Proteomics Bioinform. 2011, 4:179-183. |
| P*_luxS_* | *Bacillus licheniformis* | A hybrid promoter, combining partial coding region of *ylyb* gene with 5'-end partial region of *luxS* gene | PLoS One. 2013, 8:e56321 |
| P*_gsiB_* | *Bacillus subtilis* | Recognized by the σ^B^ RNA polymerase and activated by the mild stresses | Mol Gen Genet. 1995, 248:114-120; 1998, 258:538-545 |
| P*_yxiE_* | *Bacillus subtilis* 168 | Recognized by the σ^A^ RNA polymerase | Biochem Bioph Res Commun. 2007, 354:90-95. |
